# Age-Related Changes in Cardiac Autonomic Modulation and Heart Rate Variability in Mice

**DOI:** 10.3389/fnins.2021.617698

**Published:** 2021-05-17

**Authors:** Chiara Piantoni, Luca Carnevali, David Molla, Andrea Barbuti, Dario DiFrancesco, Annalisa Bucchi, Mirko Baruscotti

**Affiliations:** ^1^Department of Biosciences, The PaceLab and “Centro Interuniversitario di Medicina Molecolare e Biofisica Applicata”, Università degli Studi di Milano, Milan, Italy; ^2^Institute of Neurophysiology, Hannover Medical School, Hanover, Germany; ^3^Stress Physiology Lab, Department of Chemistry, Life Sciences and Environmental Sustainability, University of Parma, Parma, Italy; ^4^IBF-CNR, University of Milano Unit, Milan, Italy

**Keywords:** heart rate variability (HRV), aging, mouse model, autonomic cardiac modulation, arrhythmias (cardiac)

## Abstract

**Objective:**

The aim of this study was to assess age-related changes in cardiac autonomic modulation and heart rate variability (HRV) and their association with spontaneous and pharmacologically induced vulnerability to cardiac arrhythmias, to verify the translational relevance of mouse models for further in-depth evaluation of the link between autonomic changes and increased arrhythmic risk with advancing age.

**Methods:**

Heart rate (HR) and time- and frequency-domain indexes of HRV were calculated from Electrocardiogram (ECG) recordings in two groups of conscious mice of different ages (4 and 19 months old) (i) during daily undisturbed conditions, (ii) following peripheral β-adrenergic (atenolol), muscarinic (methylscopolamine), and β-adrenergic + muscarinic blockades, and (iii) following β-adrenergic (isoprenaline) stimulation. Vulnerability to arrhythmias was evaluated during daily undisturbed conditions and following β-adrenergic stimulation.

**Results:**

HRV analysis and HR responses to autonomic blockades revealed that 19-month-old mice had a lower vagal modulation of cardiac function compared with 4-month-old mice. This age-related autonomic effect was not reflected in changes in HR, since intrinsic HR was lower in 19-month-old compared with 4-month-old mice. Both time- and frequency-domain HRV indexes were reduced following muscarinic, but not β-adrenergic blockade in younger mice, and to a lesser extent in older mice, suggesting that HRV is largely modulated by vagal tone in mice. Finally, 19-month-old mice showed a larger vulnerability to both spontaneous and isoprenaline-induced arrhythmias.

**Conclusion:**

The present study combines HRV analysis and selective pharmacological autonomic blockades to document an age-related impairment in cardiac vagal modulation in mice which is consistent with the human condition. Given their short life span, mice could be further exploited as an aged model for studying the trajectory of vagal decline with advancing age using HRV measures, and the mechanisms underlying its association with proarrhythmic remodeling of the senescent heart.

## Introduction

This study explores the translational relevance of mouse models for the investigation of the link between cardiac autonomic changes and increased arrhythmic risk with advancing age. In humans, the natural process of aging is associated with a progressive structural and functional remodeling of the heart that predisposes elderly people to higher vulnerability to both brady- and tachyarrhythmias, causing substantial morbidity and mortality (Lakatta et al., 2001; Yazdanyar and Newman, 2009; Chow et al., 2012; Chadda et al., 2018; Curtis et al., 2018). Susceptibility to arrhythmogenesis is enhanced in the senescent heart even in the absence of apparent structural abnormalities, and this instability may depend on age-dependent modifications of both the electrical profile of cardiac cells (i.e., ion currents balance), and of cardiac autonomic nervous system (ANS) function (Jovanovic, 2006; Chow et al., 2012; Jeevaratnam et al., 2017; Chadda et al., 2018; Curtis et al., 2018). For example, basal plasma norepinephrine levels increase with age (Pfeifer et al., 1983), suggesting that sympathetic nervous activity may be elevated in elderly people and affect the electrical stability of both atria and ventricles (Shen and Zipes, 2014; Kalla et al., 2016; Curtis et al., 2018). Moreover, an age-related impairment in cardiac vagal modulation has been documented in studies reporting (i) a decline in the vagal component of heart rate variability (HRV) – a surrogate measure of ANS function – and (ii) diminished heart rate (HR) responses to blockade of muscarinic acetylcholine receptors with advancing age (Korkushko et al., 1991; Poller et al., 1997; Umetani et al., 1998; Antelmi et al., 2004; De Meersman and Stein, 2007). Therefore, the aim of this study is to assess whether the aging process in mice is characterized by similar changes in cardiac autonomic modulation and HRV, and whether these changes lead to increased vulnerability to cardiac arrhythmias.

Indeed, low vagally mediated HRV has been proposed as a prognostic marker of increased mortality and propensity to lethal ventricular arrhythmias in post-myocardial infarction and chronic heart failure patients (La Rovere et al., 2003; Frenneaux, 2004; Huikuri and Stein, 2012), and associated with increased Cardiovascular Disease (CVD) morbidity and mortality in the elderly population (Tsuji et al., 1994).

Further support to this view comes from the observation that increased vagally mediated HRV has been described in centenarians compared with old adults (Piccirillo et al., 1998; Paolisso et al., 1999), suggesting that the maintenance of a sound vagal tone may be crucial for successful aging and longevity (Hernandez-Vicente et al., 2020). It must be noted, however, that other studies have shown that increased vagally mediated HRV may also predict cardiac mortality in elderly populations (Dekker et al., 1997; de Bruyne et al., 1999). These contrasting reports may find an explanation in the fact that vagal stimulation is antiarrhythmic in the ventricles but proarrhythmic in the atria (Shen and Zipes, 2014), and may hint at a more complex interplay between patterns of cardiac ANS activity and the electrical stability of the myocardium during the aging process (Chadda et al., 2018; Winter et al., 2018).

Animal models could therefore be useful to shed light on the relation between aging of the cardiac ANS, decline in vagally mediated HRV, and proarrhythmic electrical remodeling of the heart. For example, a longitudinal study in rats reported a gradual increase in the occurrence of different types of spontaneous arrhythmias with aging, which was coupled with a decline in vagally mediated HRV and a progressive alteration of the specialized conducting system (Rossi et al., 2014). More recently, resting measures of vagally mediated HRV were found to predict vulnerability to pharmacologically induced ventricular arrhythmias in healthy adult rats (Carnevali et al., 2019), and to correlate with the severity of spontaneous ventricular tachyarrhythmias in transgenic, aged (10-month-old) mice overexpressing the β2 adrenoceptors (He et al., 2020). Previous attempts to characterize age-related changes in HRV in mice seem to suggest a decrease of HRV with aging; however, their interpretation in the context of a proper animal model for human aging presents some limitations. Indeed, one (Yaniv et al., 2016) identifies this decrease, but was carried out under anesthesia, while the other (Axsom et al., 2020), carried out in conscious and unrestrained animals maintained at laboratory temperature, reports a significant decrease of the time-domain parameters, but not of parameters of the frequency domain. The conclusion of these studies is therefore yet incomplete, and before addressing mechanistic hypothesis it is of foremost importance to ascertain whether mouse models are suitable for the cardiac ANS changes that characterize the natural process of aging in humans. Moreover, while HRV in humans is thought to be predominantly modulated by vagal influences (Goldstein et al., 2011; Billman, 2013; Reyes del Paso et al., 2013; Laborde et al., 2017), a discussion of the specificity of commonly used time- and frequency-domain indexes of HRV to capture features of cardiac ANS modulation in mice is still incomplete. This is relevant also in light of the much higher HR and respiratory rate that characterize mice (Carnevali et al., 2013) compared to humans, which might affect the proper computation of HRV. Finally, to the best of our knowledge, no studies have investigated the relationship between changes in cardiac ANS modulation and arrhythmia vulnerability with advancing age in mice.

On the basis of these considerations, the purposes of this study are threefold. First, to investigate the relative contribution of sympathetic and vagal components on HR modulation in two groups of conscious mice of different age. To this end, we measured HR and HRV parameters from Electrocardiogram (ECG) recordings performed during daily undisturbed conditions, and following exposure to pharmacological autonomic challenges. We hypothesized that resting HR would be predominantly modulated by sympathetic influences in older mice and that this would be reflected by (i) reduced HRV, (ii) larger HR responses (i.e., decreases) to sympathetic block with atenolol (β-adrenergic receptor antagonist), and (iii) lower HR responses (i.e., increases) to vagal block with methylscoplamine (muscarinic receptor antagonist) compared to younger mice. Second, to discuss the specificity of time- and frequency-domain HRV indexes to reliably reflect these autonomic changes. Anticipating that HRV would be largely modulated by vagal influences in mice, as in humans (Laborde et al., 2017), we hypothesized that both time- and frequency-domain HRV indexes would be reduced following muscarinic, but not β-adrenergic, block in younger mice, and to a lesser extent in older mice. Third, to evaluate age-related changes in the vulnerability to both spontaneous and pharmacologically induced cardiac arrhythmias. We expected a larger incidence of cardiac arrhythmias both under undisturbed resting conditions and following potent sympathetic stimulation with isoprenaline (non-selective β-adrenergic agonist) in older mice.

## Materials and Methods

### Animals

C57BL/6J mice (JAX from Charles River Europe) of two different ages (4-month-old and 19-month-old) were individually housed, kept in rooms with controlled temperature (22 ± 2°C) and humidity (60 ± 10%), and maintained in a 12/12 h light/dark cycle (light on from 7:00 to 19:00 h), with food and water *ad libitum*.

Experiments were performed in accordance with the European Community Council Directive 2010/63/UE and approved by the Italian legislation on animal experimentation (D.L. 04/04/2014, n. 26, authorization n. 141/2016). All efforts were made to reduce sample size and minimize animal suffering.

### Surgery: Transmitter Implantation

Mice were anesthetized using inhaled isoflurane (Isoflurane-VET, Merial). Anesthesia was induced by spontaneous breathing of 5% isoflurane in 100% oxygen at a flow rate of 1 L/min and then maintained at 1.5-3% isoflurane in 100% oxygen at a flow rate of 1 L/min; all animals received the analgesic Rymadil (5 mg/kg, Pfizer) and antibiotic Baytril (5.8 mg/kg, Bayer) immediately prior to transmitter implantation. Mice were then implanted with radiotelemetric transmitters (TA10ETA-F20, Data Sciences Int., St. Paul, MN, United States) for recordings of ECG (sampling frequency 2000 Hz), and locomotor activity (LOC, expressed as counts/minute) signals. The transmitter was placed in the abdominal cavity; one electrode was fixed to the dorsal surface of the xiphoid process and another electrode was placed in the anterior mediastinum close to the right atrium, according to a previously described procedure (Sgoifo et al., 1996). This electrode location guarantees high-quality ECG recordings, even during vigorous somatomotor activity. Body heat was maintained both during and immediately after surgery. Animals were given food and water post-surgery and were housed individually. Rymadil and Baytril were given to the animals for five consecutive days after surgery. Prior to the start of experimental recordings, mice were allowed to recover two weeks and re-establish normal daily rhythms of HR and LOC.

### Daily Recordings of ECG and LOC Signals

After recovery from surgery, ECG and LOC signals were recorded for 120 s every 30 min for six consecutive days, with the animals left undisturbed in their home cages. ECG and LOC signals were picked up by platform receivers (RPC-1, Data Sciences Int., St. Paul, MN, United States) and acquired via Dataquest A.R.T. (TM) Gold 4.3 acquisition system (Data Sciences Int., St. Paul, MN, United States).

### Pharmacological Autonomic Blockades

Mice were injected intraperitoneally on different days, following a rotational design, with: (i) 1 ml/kg saline solution (0.9% NaCl; control condition); (ii) 0.1 mg/kg methylscopolamine (muscarinic receptor antagonist, Sigma-Aldrich); (iii) 1 mg/kg atenolol (β1-adrenergic receptor antagonist, Sigma-Aldrich); (iv) methylscopolamine + atenolol (at the same above-indicated doses; for both vagal and sympathetic blockade). Each injection was separated by at least a 2-day washout period. Drug doses were selected on the basis of previous studies (Statello et al., 2017). ECG recordings were performed during the hour that preceded (baseline condition) and the 2 h that followed each injection. All injections were done between 14.00 and 15.00 h.

### Pharmacological Sympathetic Stimulation

Mice were injected intraperitoneally with isoprenaline (non-selective β-adrenergic agonist, Sigma-Aldrich, at a dose of 0.2 mg/kg for mimicking potent sympathetic stimulation). ECG recordings were performed during the hour that preceded (baseline condition) and the hour that followed the injection. All injections were done between 14.00 and 15.00 h.

### ECG Analysis

Initially, each raw ECG signal was visually inspected to ensure that all R-waves were correctly detected. Those parts of ECG recordings which exhibited recording artifacts were discarded without substitution and excluded from further analysis. Heart rate (reported in beats per minute, bpm) and time- and frequency-domain parameters of HRV were quantified using ChartPro 5.0 software (ADInstruments, Sydney, Australia), following the guidelines suggested by Thireau and colleagues (Thireau et al., 2008) for the assessment of HRV parameters in mice. Time-domain measures included the standard deviation of the time between normal-to-normal beats (SDNN) and the root mean squared of successive beat-to-beat interval differences (RMSSD) (No authors listed, 1996). For spectral (frequency-domain) analysis of HRV, a power spectrum was obtained with a fast Fourier transform-based method (Welch’s periodogram: 256 points, 50% overlap, and Hamming window). We considered: (i) the total power of the spectrum (TP, ms^2^), (ii) the power (ms^2^) of the low frequency band (LF, 0.15–1.5 Hz) and (HF, 1.5–5.0 Hz) bands in absolute values (ms^2^) and normalized units (n.u.), and (iii) the low frequency/high frequency ratio (LF/HF).

### Data Analysis

#### Daily Recordings of ECG and LOC Signals

Separate estimates of HR, HRV, and LOC were initially generated for each 2-min recording period and subsequently averaged as mean values of the 12 h-light and 12 h-dark phase of each recording day. These parameters were then further averaged to obtain light phase and dark phase means of the six recording days. Subsequently, to test for differences between the two groups of mice, a series of two-way ANOVAs for repeated measures were applied on HR and HRV data, with “group” as the between-subject factor (two levels: 4-month-old and 19-month-old mice) and “phase” as the within-subject factor (two levels: light and dark phases). Follow-up *post hoc* analyses were conducted using Fisher’s LSD.

#### Pharmacological Autonomic Manipulations

Each recording period was split in 2-min epochs (0–2 min, 2–4, etc.). For each epoch, separate estimates of HR and HRV were generated. Initially, to test the effects of pharmacological autonomic manipulations on HR values within each group, ΔHR values were calculated as the differences between each 2-min post-injection period and the respective mean baseline value, and then averaged for the 60-min period corresponding to the second hour that followed each injection [min 60–120; i.e., when animals had completely re-established baseline HR and HRV following stress associated with handling and injection, as shown in the saline (control) condition]. For each group, ΔHR and ΔHRV values were analyzed by means of one-way ANOVAs for repeated measures, with “autonomic manipulation” as the within subject factor [4 levels: control (saline) condition, vagal blockade, sympathetic blockade, double blockade]. Follow-up analyses were conducted using Student’s *t*-tests, with a Fisher’s correction for multiple comparisons.

#### Incidence of Cardiac Arrhythmias

Lastly, the occurrence of spontaneous and pharmacologically (isoprenaline)-induced cardiac arrhythmias was determined and quantified off-line as in Surawicz and Knilans (2008), Curtis et al. (2013), Carnevali et al. (2015). We identified and quantified the separate occurrence of sinus pauses, atrioventricular blocks, and supraventricular (SV) and ventricular (V) ectopic beats and the total number of arrhythmic events [reported as number of events per length (hours) of analyzed ECG recording] during daily undisturbed conditions and following pharmacological sympathetic stimulation with isoprenaline. Age-related changes in arrhythmia vulnerability were assessed by means of unpaired Student’s *t*-tests.

All data in figures and tables are presented as means ± SEM. All statistical analyses were performed using SPSS 24 software package (SPSS Inc. Chicago, IL) and OriginPro 2020 (OriginLab Corporation, Northampton, MA). Statistical significance was set at *p* < 0.05.

## Results

### Daily Recordings

Basal HR and HRV indexes were evaluated in 4 and 19 month-old freely moving mice to identify possible aging associated changes.

**FIGURE 1:**
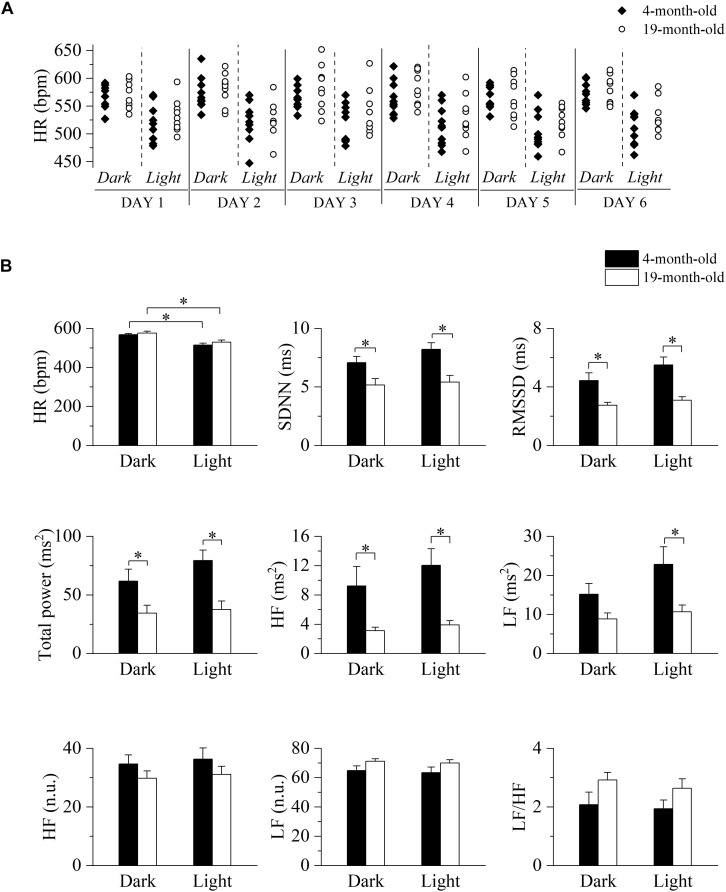
Long-term evaluation of HR and HRV in 4- and 19-month-old freely moving mice. **(A)** Time course of heart rates (HR) recorded during the 12-h light and 12-h dark phases of six consecutive days in 4-month-old (*n* = 10, filled diamonds) and 19-month-old (*n* = 9, empty circles) freely moving mice. **(B)** Mean heart rate and heart rate variability parameters evaluated from ECG traces recorded from 4-month-old and 19-month-old mice during the daily cycle (dark and light phases). Data are reported as mean ± SEM. SDNN, standard deviation of beat-to-beat intervals; RMSSD, root mean square of successive beat-to-beat interval differences; LF, low frequency; HF, high frequency; LF (n.u.), low frequency in normalized units; HF (n.u.), high frequency in normalized units. ^∗^*p* < 0.05, two-way ANOVA for repeated measures followed by Fisher’s LSD *post hoc* test.

We initially tested for the presence of a circadian oscillation of HR and to this aim we collected 2 min-long ECG traces every 30 min for six consecutive days. Heart rate values measured during light and dark phases of equal duration of the daily cycle are shown for each mouse in Figure 1A, while collective mean ± SEM data are shown in panel B (top, left) and presented in Supplementary Table 1. As expected, heart rates during the dark/active phase of the daily cycle were significantly higher than those during the light/inactive phase for each age group; comparison between age-groups in the light and dark conditions did not reveal significant differences, thus confirming that aging does not impact on basal heart rate, an observation that is consistent with literature data on humans and mice. Also, no difference in the locomotor activity was detected between the groups (Supplementary Table 1). HRV indexes, extracted from the same ECG recordings, are plotted in Figure 1B (data in Supplementary Table 1). Despite the similarity in basal HR, the 19-month-old group showed significantly lower values of SDNN, RMSSD, total power, and HF power during both phases of the daily cycle, while LF power was reduced only during the light phase. No significant differences were instead observed when the LF and HF bands were expressed in normalized units as well as in the LF/HF ratio.

### HR Responses to Pharmacological Autonomic Blockades

**FIGURE 2:**
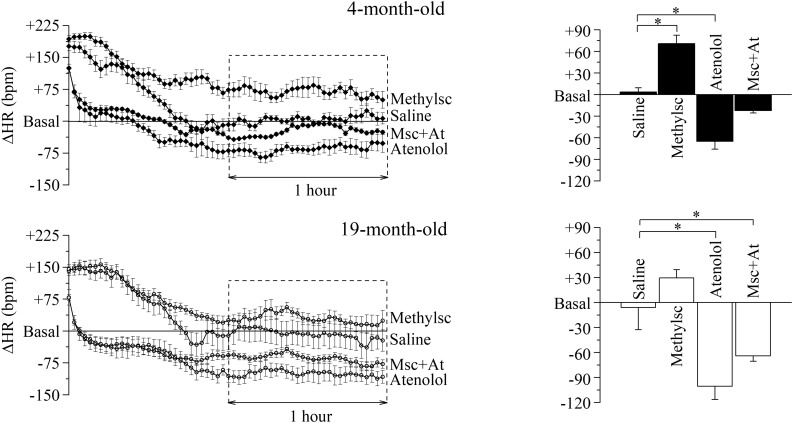
Age-related differences in the autonomic modulation of heart rate. Heart rate changes (ΔHR) after i.p. injection of vehicle (Saline) and autonomic modulator drugs (Methylscopolamine, Atenolol and Methylscopolamine + Atenolol) in 4 month-old (*n* = 10) and 19 month-old (*n* = 9) freely moving mice. The left-hand panels represent the time courses of ΔHR recorded from the moment of the injection; each point represents the mean ΔHR of a 2-min period. Saline was employed to identify the time span which is needed by the animals to recover from the handling-induced stress (60 min after the injection the HR returned similar to the mean basal HR recorded for 60 min before drugs administration, Basal). The right-hand panels report mean ΔHR values recorded during the second hour post-injection. Data are reported as mean ± SEM. ^∗^*p* < 0.05, one-way ANOVA for repeated measures followed by Fisher’s LSD *post hoc* test.

In order to investigate the influences of the Autonomic Nervous System on HR and dissect the relative contribution of each autonomic arm in the two age conditions, we evaluated the HR responses to selective pharmacological blockades of the parasympathetic and sympathetic systems (Figure 2) by i.p. injection of methylscopolamine and/or atenolol. Since HR and HRV indexes are greatly influenced by the experimental handling of the mouse, we first identified a protocol where this stress-induced experimental artifacts could be eliminated. To this aim we injected the mice with a saline solution and verified that complete recovery to the basal level (defined as the mean value measured during 1 h preceding the treatments) was attained after 1 h from the injection time. All the analyses were then carried on the data recorded during the following hour (squared dashed box in the figure). The time-courses of HR changes (ΔHR) following each autonomic manipulation are shown overlapped separately for the two age groups (Figure 2, left). Mean ± SEM ΔHR values are presented in the right panels of Figure 2 and are listed in Supplementary Table 2.

Within-group statistics yielded a significant effect of “autonomic manipulation” both in 4-month-old and 19-month-old mice. Specifically, *post hoc* analysis revealed that in 4-month-old mice vagal blockade with methylscopolamine provoked a significant increase in HR compared with the control (i.e., saline) condition (*p* < 0.05), whereas sympathetic blockade with atenolol provoked a significant decrease in HR compared with the control condition (*p* < 0.05). No significant changes in HR were observed in 4-month-old mice after double autonomic blockade (methylscopolamine + atenolol) compared with the control condition. In the 19-month-old group a significant decrease in HR was observed during sympathetic blockade (*p* < 0.05), while an increase, albeit not significant, was observed during parasympathetic deprivation. Quite interestingly, in the older group the double autonomic blockade caused a significant decrease of HR, thus revealing a substantial difference between the intrinsic and basal rate.

We also further proceeded to verify whether the effect of autonomic blockade could reveal aging associated differences. To this aim we compared heart rate differences (ΔHR) during each specific pharmacological manipulation and confirmed that both vagal and double autonomic blockade were different between the two age groups (Student’s *t*-test, *p* < 0.05), while no difference was identified during sympathetic deprivation.

### HRV Responses to Pharmacological Autonomic Blockades

**FIGURE 3:**
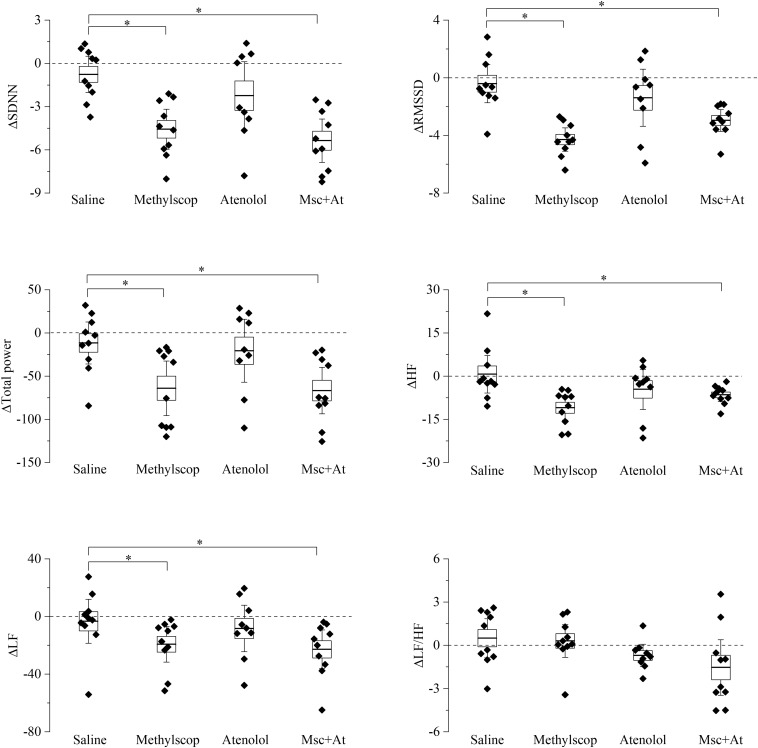
Heart rate variability parameters response to pharmacological autonomic blockade in 4-month-old mice. Changes in the heart rate variability parameters calculated from ECG traces recorded from 60 to 120 min after i.p. injection of vehicle (saline) and autonomic modulator drugs (Methylscopolamine, Atenolol, Methylscopolamine + Atenolol) in 4-month-old freely moving mice (*n* = 10). The dotted line (reference level) indicates mean basal HRV indexes recorded 60 min before drug administration. In the box chart, middle line indicates the mean value, delimiting lines of the box represent SEM and whiskers are confidence intervals at 95%. ^∗^*p* < 0.05, one-way ANOVA with repeated measures followed by Fisher’s LSD *post hoc* test.

**FIGURE 4:**
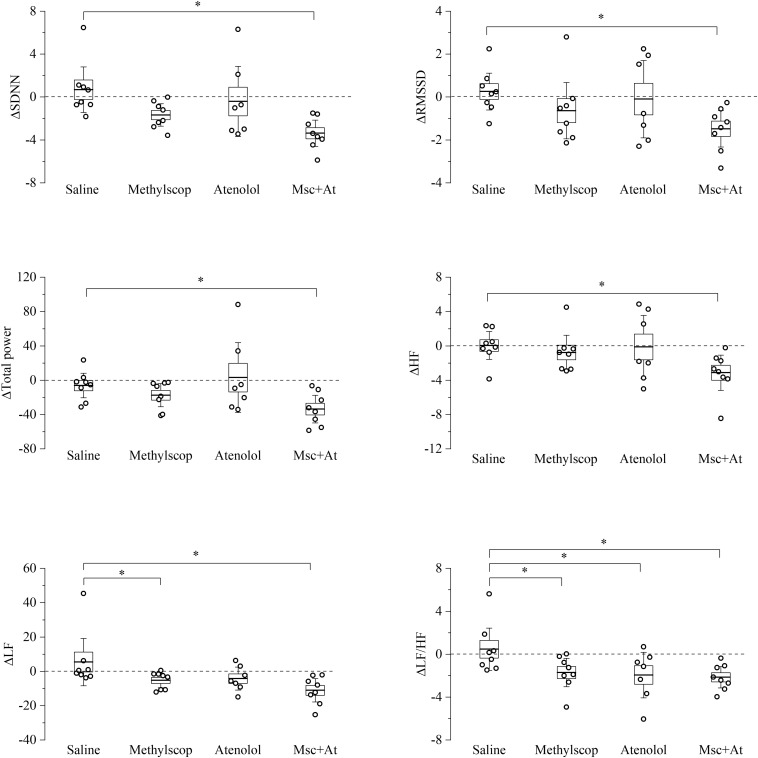
Heart rate variability parameters response to pharmacological autonomic blockade in 19-month-old mice. Changes in the heart rate variability parameters calculated from ECG traces recorded from 60 to 120 min after i.p. injection of vehicle (saline) and autonomic modulator drugs (Methylscopolamine, Atenolol, Methylscopolamine + Atenolol) in 19-month-old freely moving mice (*n* = 9). The dotted line (reference level) indicates mean basal HRV indexes recorded 60 min before drug administration. In the box chart, middle line indicates the mean value, delimiting lines of the box represent SEM and whiskers are confidence intervals at 95%. ^∗^*p* < 0.05, one-way ANOVA with repeated measures followed by Fisher’s LSD *post hoc* test.

Basal heart rate results from the complex balance of at least three main elements: intrinsic heart rate, sympathetic and parasympathetic drive. Results obtained in previous figures clearly illustrate that 4 and 19 month-old mice have similar basal heart rates, but the underlying relative contribution of the above-mentioned elements is different. To shed light on this point and better characterize the differences, HRV analysis was carried out on the ECG traces collected during selective autonomic blockades (Figures 3, 4). In this case, we calculated differences in the HRV indexes measured during each autonomic manipulation or saline injection versus those calculated in Basal condition (prior to treatments). In 4-month-old mice (Figure 3 and Supplementary Table 3), vagal blockade (methylscopolamine) and double autonomic block (methylscopolamine + atenolol) significantly reduced (*p* < 0.05) SDNN, RMSSD, total power, HF, and LF values compared with the control (saline) condition. No differences were observed in the LF/HF ratio. Interestingly, sympathetic blockade alone did not result in significant changes in any index.

In 19-month-old mice (Figure 4 and Supplementary Table 4), unexpectedly, vagal or sympathetic blockade alone did not result in significant changes in SDNN, RMSSD, total power, HF, and LF. However, significant reductions (*p* < 0.05) of all HRV parameters were observed under double autonomic blockade. In addition, differences LF/HF values were identified during sympathetic blockades, respectively.

Taken together the result of Figures 3, 4 point to a lower level of rate variability in aged mice, and this appear to be mostly associated with a decreased vagal component.

### Vulnerability to Cardiac Arrhythmias

A more rigid chronotropism (i.e., a reduction of HRV) is a pro-arrhythmic marker in humans; therefore, based on our previous findings, and with the overall aim to test whether the mouse is a reasonable experimental model to study cardiac aging, we further proceeded to verify the propensity to arrhythmias in the two age groups.

**FIGURE 5:**
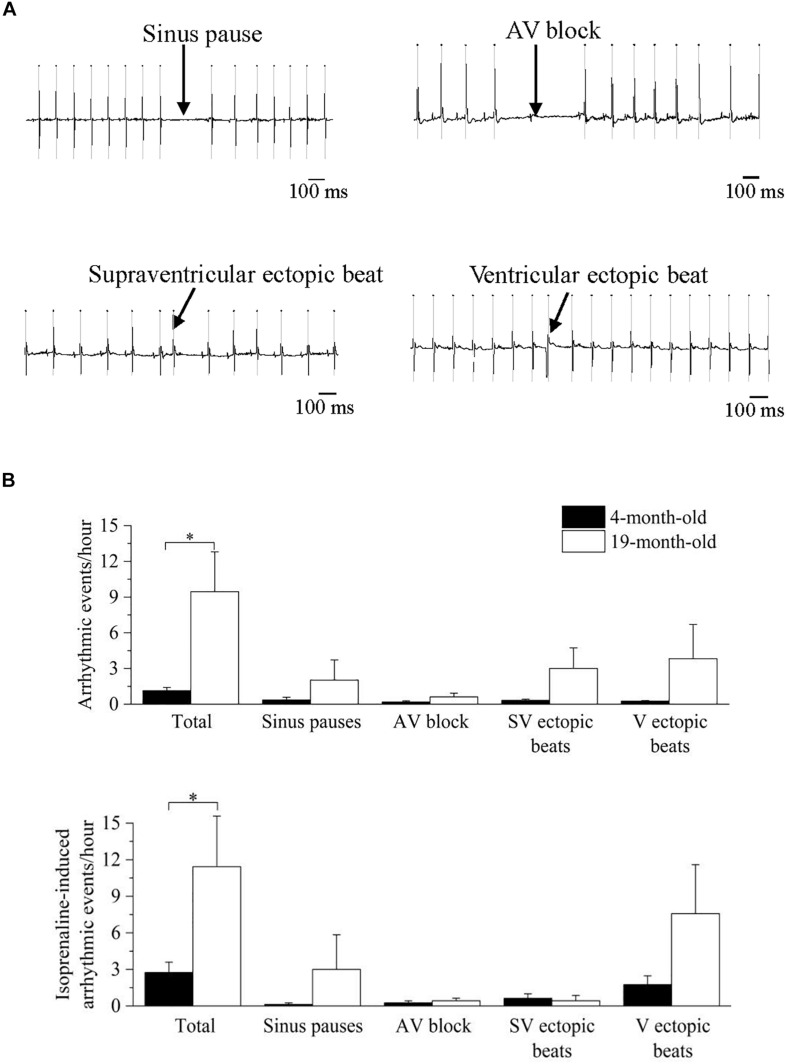
Analysis and classification of spontaneous and induced arrhythmic events occurred in 4- and 19-month-old mice. **(A)** sample ECG traces of four different types of arrhythmic events: sinus pause, atrioventricular block, supraventricular ectopic beat and ventricular ectopic beat. **(B)** mean values of the spontaneous (top) and isoprenaline-induced (bottom) total and type-specific (sinus pauses, atrioventricular block, supraventricular ectopic beats and ventricular ectopic beats) arrhythmic events. Data are reported as mean ± SEM. Comparisons were evaluated by Student’s *t*-test, ^∗^*p* < 0.05.

In Figure 5 representative recordings (panel A) and mean ± SEM cumulative data (panel B) of the arrhythmic events observed in the mice are shown. During undisturbed daily recordings (Figure 5B, top), the overall incidence of cardiac arrhythmias was significantly larger in 19-month-old-mice compared to 4-month-old mice (*p* < 0.05), without specific age-related differences in the incidence of the various arrhythmic subtypes.

Since an increased adrenergic background drive is also a well-known pro-arrhythmic condition, we repeated the analysis of arrhythmic events in the presence of adrenergic stimuli with isprenaline, and a similar larger overall incidence of cardiac arrhythmias was found in 19-month-old mice (Figure 5B, bottom). Of note, HR response to maximal ISO stimulation, measured as ΔHR was significantly smaller (*p* < 0.05 Student’s *t*-test) in 19-month-old (171.4 ± 16.0 bpm, *n* = 7) compared with 4-month-old mice (226.8 ± 14.3 bpm, *n* = 8).

## Discussion

The present study combines HRV analysis and selective pharmacological autonomic challenges to demonstrate a relative lower contribution of the vagal component on HR modulation in 19-month-old mice compared with 4-month-old mice. This age-related cardiac autonomic effect is (i) observed both during the light and dark phases of the daily cycle in conscious and freely moving mice, (ii) independent from somatomotor activity levels, (iii) compensated by a reduction in intrinsic HR, leading to a stable basal resting HR with advancing age, and (iv) associated with a larger vulnerability to both spontaneous and pharmacologically induced arrhythmias. In the next sections, we discuss the age-related changes in cardiac ANS modulation, the utility of commonly used time- and frequency-domain indexes of HRV to capture these autonomic changes in mice, and their association with arrhythmia susceptibility.

### Age-Related Changes in Cardiac ANS Modulation in Mice

Specific patterns of cardiac ANS modulation may vary across species, with possible species-typical sympathovagal balance (Evans et al., 1990; Japundzic et al., 1990; Carnevali and Sgoifo, 2014). For example, resting HR is characterized by a relatively larger vagal prevalence in humans (Tan et al., 2009) while it is generally believed that mice have a sympathetically dominated resting HR (Ishii et al., 1996; Gehrmann et al., 2000; Axsom et al., 2020), thus questioning the translational relevance of the murine model for the study of cardiac ANS-related conditions. For example, a recent study suggested that mice operate under high sympathetic drive in order to maintain a normal core temperature in standard laboratory conditions (20°C), and that when mice are acclimated to their thermoneutral zone (30°C) for 3 days, sympathetic input to the heart is greatly reduced (Axsom et al., 2020). Based on these findings, the authors conclude that in the context of a translational model that recapitulates human physiology, it is vital that mice be housed under thermoneutral conditions to allow for normal autonomic regulation (Axsom et al., 2020). However, one may argue that patterns of ANS modulation at 30°C might represent a transient physiological response to environmental alterations in mice that were born and raised at ∼20°C. Moreover, it must be noted that previous attempts to characterize age-related ANS changes in mice were performed under anesthesia (which may affect ANS function) or during short-term recordings (which do not take into account potential circadian effects on ANS function) (Yaniv et al., 2016; Axsom et al., 2020; He et al., 2020). Therefore, in our study we sought to overcome these limitations by assessing age-related changes in cardiac autonomic modulation using two different and complementary approaches: via HRV analysis of ECG signals obtained during daily recordings at standard laboratory temperature conditions (∼22°C) in conscious mice of different age (4 and 19 months old), and by evaluating HR responses to selective pharmacological autonomic blockades.

We found an age-related reduction in time- (SDNN, RMSSD) and frequency-domain (total and HF power values) indexes of HRV during both phases of the daily cycle (Figure 1B), which recapitulates human findings (Jandackova et al., 2016). Power values of the LF index was instead reduced only during daytime analysis. Importantly, we provide evidence that age-related changes in HRV are not ascribable to changes in somatomotor activity levels. Relatedly, we observed a significant increment in HR values under vagal blockade with methylscopolamine in 4-month-old, but not in 19-month-old, mice compared with the control (saline) condition (Figure 2). Taken together, these data (see below our discussion on HRV indexes) are indicative of a decline in resting cardiac vagal modulation with advancing age that typically characterizes humans (De Meersman and Stein, 2007). On the other hand, adrenergic receptor blockade with atenolol led to a decrease in the average HR in both groups with a larger, albeit not significant effect observed in the 19 month-old group. Notably, these age-related ANS changes were not reflected by concomitant changes in resting measures of HR. A potential explanation lies in the fact that the intrinsic HR (i.e., HR under double autonomic blockade) was significantly lower in 19-month-old mice compared to the younger group. It has been suggested that changes in the mechanisms intrinsic to heart pacemaker cells may compensate for the aging-associated shift in the sympathovagal balance toward a sympathetic prevalence to preserve a stable resting HR (De Meersman and Stein, 2007).

In interpreting our findings from a translational point of view, we must underline similarities and differences with the human condition. First, the fact that muscarinic receptor blockade with methylscopolamine was associated with a significant increase of the average HR in the younger group suggests that in young adult mice vagal modulation plays a significant role in the regulation of resting HR. It must be acknowledged, however, that the intrinsic HR in the young group was similar to the average resting HR, confirming that, contrary to young adult humans (Tan et al., 2009), resting HR in young adult mice is not predominantly modulated by vagal influences. Most importantly, our data indicate that mice exhibit age-related changes in cardiac ANS regulation (i.e., decline in vagal functioning) and automaticity of cardiac pacemaker cells (i.e., reduced intrinsic HR) that closely recapitulate those observed in human populations (Jose and Collison, 1970; Korkushko et al., 1991; Poller et al., 1997; Umetani et al., 1998; Antelmi et al., 2004; De Meersman and Stein, 2007). Moreover, we also found that, similar to humans (White et al., 1994), maximal β-adrenergic receptor stimulation with isoprenaline provoked a smaller HR response in the older group. This result is in agreement with a previous study documenting a reduced sensitivity of the average heart beating intervals to β-adrenergic receptor stimulation in the isolated SAN tissue of older mice (Yaniv et al., 2016). However, the correct evaluation of the response to maximal adrenergic stimuli should likely consider as a starting point the intrinsic HR and not basal HR since in this last condition adrenergic receptors may be already partly occupied. If this consideration is taken into account the maximal adrenergic excursions observed in our mice are + 224 ± 4 bpm in 4 month-old mice and + 238 ± 14 bpm in 19-month-old mice; these similar accelerations likely suggest a fully maintained adrenergic modulation of rate. Interstingly, Peters et al. (2020), also report that the maximal heart rate attainable during adrenaline stimulation decreases with age and this reduction is mainly determined by a lower intrinsic heart rate, albeit a modest decline in the sympathetic nervous function is also present. However, in another study carried out in isolated innervated hearts of aged female mice (Francis Stuart et al., 2018), the authors suggested that the decreased HR response to isoprenaline is primarily due to a decreased β−adrenergic responsiveness since, upon sympathetic nerve stimulation, atrial noradrenaline content was similar in aged and young hearts, thus suggesting preserved sympathetic nerve density with advancing age (Francis Stuart et al., 2018). In contrast, decreased responsiveness to sympathetic nerve stimulation in the ventricles of aged female mice seemed to be primarily due to nerve degeneration, since β−adrenergic responsiveness was preserved, but sympathetic nerve density and noradrenaline content were reduced (Francis Stuart et al., 2018).

### Specificity of HRV Indexes to Capture Age-Related Changes in Cardiac ANS Modulation in Mice

HRV can be separated into various components, reflecting ANS influence on cardiac control, and its analysis has become a popular approach in several investigational domains, both in human and animal research (Berntson et al., 1997; Rowan et al., 2007; Laborde et al., 2017). In humans, the SDNN is thought to reflect all the cyclic components responsible for variability, and the SDNN squared is equivalent to the variance (total power) when viewed in the frequency domain (Shaffer and Ginsberg, 2017). The RMSSD and the HF component of HRV detect quick beat-to-beat fluctuations in a heart period time series, primarily reflecting vagal modulation (Laborde et al., 2017). In this study, we confirm the reliability of RMSSD and HF power values to assess vagally mediated HRV since in 4 month-old mice we observe a substantial reduction of these indexes following muscarinic receptor blockade with methylscopolamine (Figure 3). Relatedly, vagal blockade, but not sympathetic blockade, modulates SDNN and total power values, suggesting that a prevalent vagal influence mediates HRV in mice. This finding well correlates with the evidence that vagal block in young significantly modifies heart rate in this age group. Notably, the effect of vagal blockade on HRV indexes was significant in 4-month-old but not in 19-month-old mice (Figures 3, 4) in agreement with the above discussed decline in resting cardiac vagal functioning in the older group. Furthermore, although the LF component of HRV has been theorized to represent both sympathetic and vagal influences (Goldstein et al., 2011; Reyes del Paso et al., 2013), findings of reduced LF values following vagal, but not sympathetic, blockade in the young group suggest that the LF band [0.15–1.5 Hz in this study (Thireau et al., 2008)] captures predominantly vagal influences in mice. This finding has two important implications for the use of HRV in mice: (i) normalized LF and HF units, which report frequency power proportional to the total observed power, may not discriminate between different levels of resting cardiac vagal modulation, (ii) the LF to HF ratio should not be interpreted as an index of sympathovagal balance in mice, as already pointed out in humans (Heathers, 2012; Billman, 2013; Laborde et al., 2017). On the other hand, differences in resting measures of RMSSD and HF power values between the two age groups well support our pharmacological evidence of reduced cardiac vagal modulation with advancing age.

In the light of these considerations, we advocate the use of vagally mediated HRV indexes (i.e., RMSSD and HF power values) for capturing the decline in cardiac vagal modulation in mouse models of aging. However, it must be noted that while HRV alterations are often ascribed solely to changes in ANS signaling, age-related changes in HRV in mice have also been associated with deterioration of autonomic neuronal receptor signaling and mechanisms intrinsic to heart pacemaker cells (Yaniv et al., 2016).

### Age-Related Changes in the Vulnerability to Cardiac Arrhythmias

Reduced vagally mediated HRV has been proposed as a prognostic marker of increased mortality and propensity to lethal ventricular arrhythmias in cardiac patients (La Rovere et al., 2003; Frenneaux, 2004; Huikuri and Stein, 2012), and associated with increased CVD morbidity and mortality in the elderly (Tsuji et al., 1994).

In this study 19-month-old mice exhibited, alongside reduced vagally mediated HRV, a larger vulnerability to both spontaneous and pharmacologically-(isoprenaline-) induced cardiac arrhythmias compared to 4-month-old mice (Figure 5). Arrhythmias were both of supraventricular (e.g., sinus pauses, supraventricular ectopic beats) and ventricular (ventricular ectopic beats) origin, with no clear chamber prevalence. It must be acknowledged that the incidence of arrhythmias was relatively modest even in the older group. In fact, one of the major limitations of this study is that we used only two age groups, with the older group likely representing an early phase of the aging process in this mouse strain. Therefore, we cannot exclude that arrhythmogenesis would be more evident in older mice. Interestingly, a previous study reported a higher incidence of ventricular arrhythmias in aged female mice following rapid ventricular pacing, but not after sympathetic nerve stimulation (Francis Stuart et al., 2018). Therefore, age−related sex differences in arrhythmia vulnerability with advancing age is an interesting area for future work. Notably, in humans premature atrial and ventricular complexes are frequently present in the healthy elderly population, even in the absence of apparent structural abnormalities (Chow et al., 2012; Chadda et al., 2018; Curtis et al., 2018). For example, in the Cardiovascular Health Study (Manolio et al., 1994), 24-h ambulatory monitoring in 60- to 85-year-old healthy individuals showed that 86% of patients had premature atrial complexes, with 26% having > 36 premature atrial complexes/h. Premature ventricular complexes were found in 82% of elderly subjects, including runs of non-sustained ventricular tachycardia. Importantly, a high burden of premature ventricular complexes was associated with increased left ventricular systolic dysfunction, incident heart failure, and mortality among the participants of this study (Dukes et al., 2015). However, despite promising and preliminary results on the utility of vagally mediated HRV indexes for predicting arrhythmic risk in rodents (Carnevali et al., 2019; He et al., 2020), we believe it is premature to assume that decreased vagally mediated HRV in aged mice can be regarded as a negative prognostic indicator of CVD risk. Nevertheless, the preliminary association found in this study between decline in cardiac vagal functioning and increased vulnerability to arrhythmias with advancing age encourages future mouse research aimed at investigating the causal role of age-related perturbations in ANS in the proarrhythmic electrical remodeling of the heart.

## Conclusion

The present study documents an age-related impairment in cardiac vagal modulation and HRV in mice, which is coupled with a larger vulnerability to cardiac arrhythmias. These results should be interpreted within the context of their limitations. Besides the use of only two age groups, we must acknowledge that HRV is influenced by several factors that were not considered here, including respiratory activity and blood pressure. For example, in rodents, the respiratory pattern consists of normal (eupnoea) or rapid (tachypnoea) breathing, intermingled with periods of sniffing of variable intensity and duration (Carnevali et al., 2013). While controlling for respiration is a long debated issue within human HRV research (Laborde et al., 2017), it would be interesting to test the extent to which different respiratory behaviors influence HRV indexes in mice.

Furthermore, an important component of short- and long-term blood pressure regulation is the arterial baroreflex, which plays a central role particularly in modulating sympathetic and vagal control of the heart and peripheral vasculature to respond to shifting metabolic demands and maintenance of a stable blood pressure (Guyenet, 2006; Reyes del Paso et al., 2006). Given that aging in humans is associated with decreased cardiovagal baroreflex sensitivity (Monahan, 2007), future mouse studies should combine HRV and blood pressure monitoring to obtain a more complete picture of the cardiac autonomic changes that characterize the aging process in mice. Importantly, while one of the major advantages of using mice as an aged model is their short lifespan, available research, including the present investigation, is mostly cross-sectional (Yaniv et al., 2016; Bennett et al., 2018). Consequently, these results warrant future longitudinal studies adopting HRV measures to investigate the trajectory of vagal decline with advancing age and its causal relationship with cardiac proarrhythmic remodeling in conscious mice.

Lastly, since autonomic dysfunction can contribute to the physiological decline of cognitive functions linked to the aging process (Murman, 2015; Forte et al., 2019), it would be interesting to evaluate the extent to which mouse models can contribute to the development of theoretical models established in humans with HRV that connect the heart and brain via the vagus nerve (Thayer et al., 2009; Smith et al., 2017).

## Data Availability Statement

The original contributions presented in the study are included in the article/Supplementary Material, further inquiries can be directed to the corresponding author.

## Ethics Statement

All animal procedures performed in this study were carried out in accordance with the guidelines of the care and use of laboratory animals established by the Italian and UE laws (D. Lgs no. 2014/26, 2010/63/UE), the experimental protocols were approved by the Animal Welfare Committee of the Universitá degli Studi di Milano and by the Italian Minister of Health (protocol number 141/2016).

## Author Contributions

CP, LC, and MB: conceived and designed the research. CP and LC: acquired the data. CP, LC, DM, and MB: performed statistical analysis and drafted the manuscript. CP, LC, DM, ABu, DD, ABa, and MB: made critical revision of the manuscript for key intellectual content. DD and MB: handled funding and supervision. All authors contributed to the article and approved the submitted version.

## Conflict of Interest

The authors declare that the research was conducted in the absence of any commercial or financial relationships that could be construed as a potential conflict of interest.
