# Supplementary material for: Age-Related Changes in Cardiac Autonomic Modulation and Heart Rate Variability in Mice
Source: Front Neurosci. 2021 May 17;15:617698. doi: 10.3389/fnins.2021.617698 (PMC8168539; doi:10.3389/fnins.2021.617698)
Supplement: Supplementary file 1 [file Data_Sheet_1.PDF]

Age-related changes in cardiac autonomic modulation and heart rate variability in mice

Running Title: HRV and aging in mice

Chiara Piantoni[1,2 *], Luca Carnevali[3 *], David Molla[1], Andrea Barbuti[1], Dario DiFrancesco[1,4], Annalisa Bucchi[1], Mirko Baruscotti[1$]

1 Department of Biosciences, The PaceLab and "Centro Interuniversitario di Medicina Molecolare e Biofisica Applicata", Università degli Studi di Milano, via Celoria 26, 20133 Milano, Italy.

2 Institute of Neurophysiology, Hannover Medical School, Carl-Neuberg-Str.1, 30625 Hannover, Germany.

3 Stress Physiology Lab, Department of Chemistry, Life Sciences and Environmental Sustainability, University of Parma, Parma, Italy

4 IBF-CNR, University of Milano Unit, via Celoria 26, 20133 Milano, Italy

[*] These authors have contributed equally to this work and share first authorship

[$] Corresponding author:

Mirko Baruscotti, University of Milan, Department of Biosciences, via Celoria 26, 20133 Milano, Italy. Tel: +39 02 50314939, Email: mirko.baruscotti@unimi.it

Supplementary Material

Table S1. HR values and HRV parameters measured in dark and light phases of the circadian cycle for the two groups of mice (4- and 19-month-old).

PARAMETER	CONDITION	AGE	
		4-month-old	19-month-old
HR (bpm)	Dark	567.8±6.54	576.12±9.85
	Light	514.7±9.54	529.34±10.56
LOC (cpm)	Dark	4.46±0.83	3.81±0.52
	Light	2.20±0.42	2.33±0.41
SDNN (ms)	Dark	7.08±0.52	5.17±0.54
	Light	8.22±0.55	5.42±0.57
RMSSD (ms)	Dark	4.44±0.52	2.75±0.2
	Light	5.5±0.55	3.09±0.24
Total power (ms^2)	Dark	61.75±10.21	34.41±6.73
	Light	79.32±9	37.58±7.33
HF power (ms^2)	Dark	9.23±2.65	3.1±0.47
	Light	12.05±2.27	3.9±0.6
LF power (ms^2)	Dark	15.16±2.8	8.87±1.52
	Light	22.81±4.5	10.68±1.73
LF/HF	Dark	2.07±0.43	2.92±0.24
	Light	1.94±0.30	2.64±0.31

Table S2. Mean±SEM ΔHR values (treatment – basal) obtained in the presence of different autonomic pharmacological blockades.

PARAMETER	CONDITION	AGE	
		4-month-old	19-month-old
ΔHR	Saline	3.59±5.69	-6.04±26.47
	Methylsc	70.73±11.67	29.68±9.66
	Atenolol	-65.11±10.75	-100.47±15.71
	Msc+At	-22.39±3.39	-64.06±6.42

Table S3. Mean±SEM ∆HRV indexes (treatment – basal) measured during autonomic pharmacological blockades in 4-month-old mice.

PARAMETER	CONDITION	AGE
		4-month-old
SDNN (ms)	Saline	-0.76±0.56
	Methylscop	-4.57±0.61
	Atenolol	-2.24±1.02
	Msc+At	-5.37±0.67
RMSSD (ms)	Saline	-0.41±0.59
	Methylscop	-4.29±0.36
	Atenolol	-1.39±0.86
	Msc+At	-2.96±0.34
Total power (ms^2)	Saline	-11.68±10.73
	Methylscop	-64.02±13.93
	Atenolol	-20.61±15.83
	Msc+At	-66.74±11.89
HF power (ms^2)	Saline	0.65±2.88
	Methylscop	-10.96±1.89
	Atenolol	-4.59±3.04
	Msc+At	-6.5±1.02
LF power (ms^2)	Saline	-3.3±6.72
	Methylscop	-19.22±5.46
	Atenolol	-8.3±7.01
	Msc+At	-22.77±5.95
LF/HF	Saline	0.49±0.6
	Methylscop	0.3±0.51
	Atenolol	-0.7±0.33
	Msc+At	-1.54±0.85

Table S4. Mean±SEM ∆HRV indexes (treatment – basal) measured during autonomic pharmacological blockades in 19-month-old mice.

PARAMETER	CONDITION	AGE 19-month-old
SDNN (ms)	Saline	0.68±0.9
	Methylscop	-1.68±0.44
	Atenolol	-0.42±1.34
	Msc+At	-3.38±0.52
RMSSD (ms)	Saline	0.25±0.36
	Methylscop	-0.64±0.56
	Atenolol	-0.1±0.74
	Msc+At	-1.48±0.36
Total power (ms^2)	Saline	-6.21±6.07
	Methylscop	-17.43±5.68
	Atenolol	3.09±16.58
	Msc+At	-33.56±6.8
HF power (ms^2)	Saline	0.04±0.69
	Methylscop	-0.77±0.85
	Atenolol	-0.12±1.5
	Msc+At	-3.13±0.87
LF power (ms^2)	Saline	5.38±5.81
	Methylscop	-5.28±1.79
	Atenolol	-4.28±2.74
	Msc+At	-11.06±2.89
LF/HF	Saline	0.46±0.83
	Methylscop	-1.71±0.56
	Atenolol	-1.94±0.87
	Msc+At	-2.16±0.42
